# Preparation and Performance Characterization of Thixotropic Gelling Materials with High Temperature Stability and Wellbore Sealing Properties

**DOI:** 10.3390/polym17243343

**Published:** 2025-12-18

**Authors:** Yingbiao Liu, Xuyang Yao, Chuanming Xi, Kecheng Liu, Tao Ren

**Affiliations:** Oil Production Technology Research Institute of PetroChina Xinjiang Oilfield Company, Karamay 834000, China

**Keywords:** lost circulation, wellbore containment, thixotropic gel, plugging mechanism

## Abstract

In response to the requirements of wellbore plugging and lost circulation control, this study designed and prepared a new type of thixotropic polymer gel system. The optimal formula was obtained through systematic screening of the types and concentrations of high molecular polymers, cross-linking agents, flow pattern regulators, and resin curing agents. Comprehensive characterization of the gel’s gelling performance, thixotropic properties, high-temperature stability, shear resistance, and plugging capacity was conducted using methods such as the Sydansk bottle test, rheological testing, high-temperature aging experiments, plugging performance evaluation, as well as infrared spectroscopy, nuclear magnetic resonance, and thermogravimetric analysis, and its mechanism of action was revealed. The results show that the optimal formula is 1.2% AM-AA-AMPS terpolymer + 0.5% hydroquinone + 0.6% S-Trioxane + 0.8% modified montmorillonite + 14% modified phenolic resin. This gel system has a gelling time of 6 h, a gel strength reaching grade H, and a storage modulus of 62 Pa. It exhibits significant shear thinning characteristics in the shear rate range of 0.1~1000 s^−1^, with a viscosity recovery rate of 97.7% and a thixotropic recovery rate of 90% after shearing. It forms a complete gel at a high temperature of 160 °C, with a dehydration rate of only 8.5% and a storage modulus retention rate of 80% after aging at 140 °C for 7 days. Under water flooding conditions at 120 °C, the converted pressure-bearing capacity per 100 m reaches 24.0 MPa. Mechanism analysis confirms that the system forms a stable composite network through the synergistic effect of “covalent cross-linking—hydrogen bonding—physical adsorption”, providing a high-performance material solution for wellbore plugging in high-temperature and high-salt environments.

## 1. Introduction

Lost circulation during oil and gas drilling operations represents a prevalent and costly technical challenge, leading to significant non-productive time, increased drilling fluid costs, and potential well control risks [1,2,3]. The efficacy of lost circulation control (LCC) fundamentally hinges on the performance of plugging materials, which must rapidly form a stable, pressure-bearing seal within complex downhole environments characterized by high temperature, high salinity, and multi-scale fracture networks [4,5,6].

Existing LCC technologies often face limitations under such demanding conditions. Bridging plugging materials, including particulate and fibrous slurries, are widely used due to their cost-effectiveness and ease of deployment [7,8,9]. However, their sealing efficiency is highly dependent on fracture width, with diminishing returns observed in larger fractures. Laboratory-scale investigations using modified PPT equipment have demonstrated that multi-sized rigid particles can enhance plugging effectiveness in simplified linear fractures by improving particle packing density and interlocking stability [10]. Despite such optimizations, field data consistently show a significant drop in success rates when attempting to seal fractures exceeding 3 mm in width. This limitation arises from insufficient bridging capability and poor adaptability to irregular fracture geometries, leading to unstable plug formation and frequent failure under cyclic loading. Under fluctuating downhole pressures, even optimally designed static plugging formulations struggle to maintain integrity, particularly when subjected to pressure cycles below 8 MPa—a common scenario in fractured tight gas reservoirs [11,12]. These findings underscore the mechanistic vulnerability of granular LCMs to dynamic stress conditions, which compromise long-term zonal isolation [13].

Chemical gels, such as those cross-linked with metal ions (e.g., Cr^3+^) or organic monomers (e.g., phenol-formaldehyde), present a potential solution for adaptive sealing [14,15]. For instance, chromium-based gels exhibit thermally induced syneresis above 120 °C, leading to dehydration and loss of mechanical integrity, while also posing significant environmental risks due to the leaching of toxic heavy metals into subsurface formations [16]. Nevertheless, they are frequently hampered by inherent drawbacks. Metal-crosslinked gels can suffer from syneresis (dehydration) at temperatures exceeding 120 °C and pose environmental concerns due to heavy metal leaching [17]. Conversely, gels relying on covalent cross-linking often lack sufficient thixotropy, resulting in slow recovery times (>10 min) that fail to meet the operational requirement for instantaneous gelation upon cessation of pumping [18]. Recently, advanced systems like epoxy resins have demonstrated high strength, but their high viscosity and cost present challenges for field application, particularly in deep wells with narrow annuli [19,20]. Consequently, there is a pressing industry need for novel plugging materials that simultaneously fulfill three key criteria: pumpable shear-thinning behavior, rapid thixotropic recovery after pumping stops, and long-term stability at temperatures exceeding 160 °C.

Thixotropic polymer gels, characterized by their reversible shear-thinning and structure-recovery rheology, offer a promising avenue to address these challenges. Classic studies on thixotropy mechanisms by Ma et al. [21] highlighted that reversible network reconstruction is the core of thixotropic behavior, providing a theoretical basis for designing downhole plugging materials. For high-temperature scenarios, Wang et al. [22] reported phenol-formaldehyde gels stable at 180 °C but with poor pumpability due to excessive cross-linking, while Liu et al. [23] developed nano-SiO_2_ reinforced gels for HTHS reservoirs, yet their thixotropic recovery time (>60 s) failed to meet field requirements. In terms of environmental safety, Bhuyan et al. [24] emphasized that heavy metal-free cross-linkers are a prerequisite for large-scale application, which aligns with the design concept of our system, avoiding Cr^3+^ and other toxic components. For instance, Bai et al. [25] designed a double-network self-healing gel that maintains good modulus retention at 140 °C, though its thixotropic recovery rate remains suboptimal. The incorporation of nanomaterials, such as nano-SiO_2_, has been explored to enhance thermal stability and salinity tolerance, yet often at the expense of rapid recovery kinetics [26]. Other strategies include delayed cross-linking systems for controllable gelation times [27] and the investigation of cross-linker concentration effects on thixotropic performance [28,29]. Research by [30] on polyurethane-based gels further highlights the complex interplay between temperature, recovery speed, and gel stability.

Domestically, researchers have also made considerable strides. Modifications with antioxidants and complexing agents have been shown to significantly improve the high-temperature stability of polymer gels [31]. Thixotropic self-healing additives that regulate supramolecular structures via shear action have been developed to enhance plugging efficiency in drilling operations [32]. Despite these advancements, current thixotropic gel systems still face several bottlenecks. These include severe dehydration and network degradation at ultra-high temperatures (>160 °C), structural weakening due to ion shielding in high-salinity and high-calcium environments, inefficient thixotropic recovery (recovery time > 30 s), and challenges in process scale-up and cost-effectiveness [33]. These limitations hinder their application in deep, ultra-deep, and high-temperature-high-salinity (HTHS) oil and gas reservoirs. Some gels have slowed thixotropic recovery, unable to meet rapid response demands in practical applications [34]. Adaptive design for complex environments (high temperature, high salinity, high pollution) remains challenging, with long-term stability and reliability needing improvement [35]. Meanwhile, most existing preparation methods involve complex processes and high costs, limiting large-scale industrial application [36]. Williams [37] further pointed out that viscoplasticity and wall slip are critical factors affecting the pumpability of thixotropic gels, but few studies have systematically addressed these issues for high-temperature plugging systems—this is also a key gap our study aims to partially fill.

Therefore, to overcome the existing limitations, this study develops a novel thixotropic gel system based on an AM-AA-AMPS terpolymer skeleton, innovatively incorporating a modified phenolic resin curing agent and a modified montmorillonite flow regulator. Through systematic component screening and optimization, we aim to construct a synergistically reinforced composite network via “covalent cross-linking—hydrogen bonding—physical adsorption”. This work comprehensively evaluates the formulated system in terms of its gelling performance, thixotropic characteristics, high-temperature stability, shear resistance, and plugging capacity. Furthermore, the structure-performance relationship is elucidated using FTIR, NMR, and TGA, providing a high-performance material solution and theoretical foundation for reliable wellbore sealing in challenging HTHS reservoirs.

## 2. Experimental Section

### 2.1. Experimental Materials and Instruments

Acrylamide (AM), acrylic acid (AA), 2-acrylamido-2-methylpropanesulfonic acid (AMPS), analytical grade, purchased from Shanghai Aladdin, Shanghai, China, were copolymerized in a mass ratio of 5:3:2 to synthesize a terpolymer; formaldehyde, S-Trioxane, hexamethylenetetramine (HMTA), analytical grade, purchased from Tianjin Kemiou, Tianjin, China; hydroxyethyl cellulose (HEC), phenol, catechol, resorcinol, analytical grade, purchased from Shanghai Macklin, Shanghai, China; modified attapulgite, modified montmorillonite, purchased from Shandong Runde Biotechnology Co., Ltd., Xintai City, China; modified phenolic resin, purchased from Jiangmen Kunyi Resin Materials Technology Co., Ltd., Jiangmen, China.

The AM-AA-AMPS copolymer (P1) was synthesized via aqueous solution radical polymerization using an ammonium persulfate-sodium bisulfite initiator system (1:1 mass ratio, total addition amount: 0.8% of total monomer mass). The polymerization was conducted at 60 °C with a stirring speed of 300 rpm for 4 h. The product was obtained by ethanol precipitation, washing, and vacuum drying at 60 °C for 24 h. Using the aqueous solution polymerization method, with a mass ratio of AM to MA of 7:3, potassium persulfate as the initiator (added at 0.5%), polymerization temperature of 50 °C, and reaction time of 3 h, P2 (AM-MA binary copolymer) was prepared. P3 is a commercially procured product (Shanghai McLean Biochemical Technology Co., Ltd., Shanghai, China). According to the supplier’s technical specifications, it has a number-average molecular weight of 2.5 × 10^6^ and a hydroxyl content of 38%. The raw materials used in this system are all common industrial materials in the petrochemical field, with suppliers capable of providing large-scale supplies. The preparation process involves stirring and mixing in an aqueous solution, followed by gelation at a constant temperature, requiring no special equipment. It aligns with existing chemical production processes and possesses potential for industrial scale-up.

HAAKE Mars 60 rheometer, manufactured by Thermo Fisher Scientific (Karlsruhe, Germany), was used to determine the apparent viscosity and thixotropic loop area of materials at specific shear rates; DHG-9070A constant temperature blast drying oven, produced by Shanghai Jinghong Experimental Equipment Co., Ltd., Shanghai, China, was used to evaluate gelling time and high-temperature aging stability; FTIR-7600 Fourier transform infrared spectrometer, made by Shanghai Precision Scientific Instrument Co., Ltd., Shanghai, China, was employed to analyze the characteristics of functional groups; TGA2-SF thermogravimetric analyzer, produced by Mettler-Toledo (Greifensee, Switzerland), was used to determine the thermogravimetric curve; high-temperature and high-pressure plugging device, manufactured by Nantong Xinhua Cheng Scientific Research Instrument Co., Ltd., Nantong, China, with a maximum temperature of 200 °C and pressure of 30 MPa, was used for the plugging efficiency test of sand-filled pipe models; AVANCE 400 MHz nuclear magnetic resonance spectrometer, produced by Bruker (Zurich, Switzerland), with DMSO-d_6_ as the solvent, was used for ^13^C NMR analysis.

### 2.2. Experimental Methods

#### 2.2.1. Evaluation of Gelling Time and Strength

The sealed pressure-resistant bottles were placed in a constant-temperature blast drying oven with a preset temperature, allowing cross-linking reactions between different types of polymers and cross-linking agents to occur under constant temperature conditions. The main method used was the Sydansk bottle test, which judges the gelation dynamics by observing the changes in the flow state of the gel over time. The time when the gel strength in the pressure-resistant bottle reaches grade D is recorded as the initial gelling time, and the time when the gel strength no longer changes under high-temperature conditions is recorded as the gelling time (GT). All experimental data represent the average results of three experiments with errors not exceeding 10%.

#### 2.2.2. Rheological Strength Testing

The polymer gel systems obtained under different gelation conditions were analyzed using the plate test system of a HAAKE Mars 60 rheometer, with a rotor model P35 and a plate gap of 0.052 mm. By means of dynamic rheological testing, the gelled systems were subjected to shear mode and oscillation mode analyses. The frequency was fixed at 1 Hz, the shear rate was increased from 0.1 s^−1^ to 100 s^−1^, and the shear stress was increased from 0.1 Pa to 100 Pa. All rheological data points reported are the average of three replicate measurements. Error bars representing the standard deviation are included in the respective figures, and the relative error for all averaged values was within 10%, confirming the good reproducibility of the measurements. Limitations of rheological testing: This study did not directly measure the yield stress of the gel system (a key parameter for evaluating pumpability-plugging transition) due to the lack of a Particle Image Velocimetry (PIV) module and stress-ramp function on the current HAAKE Mars 60 rheometer. Additionally, wall slip effects—common in polymer-clay composite gels [38]—were not corrected, as multiple rheometer geometries (e.g., capillary, Couette) required for slip correction were unavailable. These limitations may lead to slight deviations in the quantitative interpretation of viscosity and modulus data; future work will use PIV-equipped rheometers and multi-geometry tests to eliminate these artifacts.

#### 2.2.3. Determination of High-Temperature Stability

The long-term stability of polymer gel systems was evaluated semi-quantitatively by the gel dehydration rate. The pressure-resistant bottles, after investigating the gelation effect, were placed in a constant-temperature blast oven at 120–160 °C. After gelation, the pressure-resistant bottles were taken out every 3 days, and the mass of the gel after water removal was weighed with a balance to calculate the dehydration rate S of the gel. The dehydration rate is the ratio of the water loss of the gel to the mass of the initial gel. All experimental data represent the average results of three experiments with errors not exceeding 10%.

#### 2.2.4. FTIR Characterization

The molecular structure of the gel was characterized using a Fourier transform infrared spectrometer. The dried gel particles and potassium bromide (KBr) were placed together in a mortar and ground into powder. The mixed powder was then pressed into a tablet under high pressure, where the total mass of the gel system powder accounted for 1.0% of the KBr powder. The scanning range was set to 400–4000 cm^−1^, and the number of scans was 35. All experimental data represent the average results of three experiments with errors not exceeding 10%.

#### 2.2.5. Thermogravimetric Analysis (TGA)

The thermal stability of the gel molecular structure was measured using a thermogravimetric analyzer (TGA). First, the instrument was turned on for preheating, and parameters such as experimental temperature and heating rate were adjusted; then an empty crucible was placed, and the mass was reset to zero; finally, an appropriate amount of sealant was placed in the instrument. For all samples, the test was conducted in a nitrogen atmosphere with a nitrogen flow rate of 20 cm^3^/min, the measurement pressure was 300 Pa, the flow rate was 20 cm^3^/min, the heating rate was 10 °C/min, and the temperature range was 30–600 °C. All experimental data represent the average results of three experiments with errors not exceeding 10%.

#### 2.2.6. Nuclear Magnetic Resonance (NMR)

Nuclear Magnetic Resonance (NMR) analysis mainly studies the absorption of radiofrequency radiation by atomic nuclei with magnetic moments in a magnetic field. It can perform qualitative analysis on the composition and structure of most organic and inorganic substances, and can also conduct quantitative analysis in some cases. In a strong magnetic field, atomic nuclei will undergo energy level splitting, and when absorbing external electromagnetic radiation, nuclear energy level transitions will occur, which is the so-called nuclear magnetic resonance phenomenon. After putting the test sample into an NMR tube and placing it in the nuclear magnetic resonance spectrometer, adjust various parameters of the instrument to carry out nuclear magnetic resonance spectrum testing on polymers and polymer gel systems. All experimental data represent the average results of three experiments with errors not exceeding 10%.

#### 2.2.7. Plugging Performance Evaluation

The plugging effect of the gel plugging agent was tested using a high-temperature and high-pressure plugging test device. A sand-filled pipe model with an inlet and outlet diameter of 6 mm, a length of 50 cm, and an inner diameter of 38 mm was used, and the pumping rate of slurry mixing water was 5 mL/min. The breakthrough pressure is related to the gel strength: the higher the strength, the higher the breakthrough pressure. The plugging capacity of the gel system was reflected by testing the breakthrough pressure of the gel at different times. All experimental data represent the average results of three experiments with errors not exceeding 10%.

## 3. Result and Discussion

### 3.1. Preparation of Thixotropic Polymer Gel System

**Figure 1:**
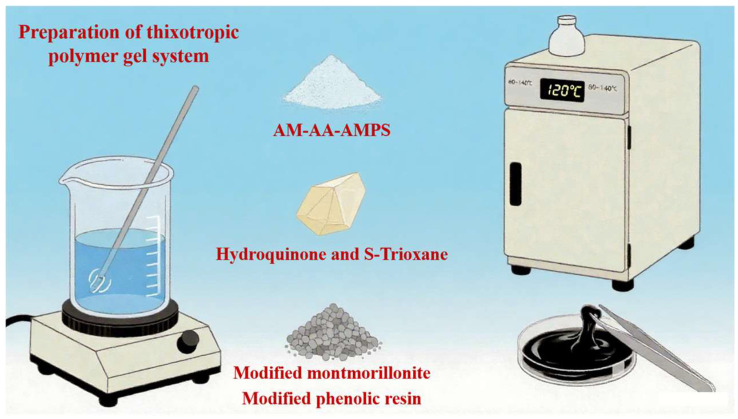
Preparation of polymer gel system.

The thixotropic polymer gel system consists of a terpolymer (AM-AA-AMPS), a cross-linking agent (S-Trioxane-hydroquinone cross-linking system), a flow pattern regulator (modified montmorillonite), and a resin curing agent (modified phenolic resin). A certain amount of deionized water was added to a beaker, and modified phenolic resin was added under magnetic stirring until it was completely dissolved. Then, a certain amount of terpolymer, cross-linking agent, and modified montmorillonite were added in sequence, and stirred for 30 min to obtain a thixotropic polymer gel solution. The prepared gelation solution (about 25 mL) was slowly transferred into a pressure-resistant bottle and placed in a constant-temperature blast oven at 80–140 °C for gel formation. The reaction pathway is illustrated in Figure 1.

#### 3.1.1. Optimization of Polymer Type and Concentration

As the framework of the thixotropic gel system, polymers contain active functional groups in their molecular structures (such as amide groups, carboxyl groups, and sulfonic acid groups), which are the core for reacting with cross-linking agents to form a three-dimensional network and directly affect gel strength, thixotropic properties, and high-temperature stability [39]. In this study, three candidate polymers were selected for screening: P-1 is an acrylamide (AM)-acrylic acid (AA)-2-acrylamido-2-methylpropanesulfonic acid (AMPS) terpolymer with a molecular weight of 5–7 million Daltons, a carboxyl group content of 12%, and a sulfonic acid group content of 15%. Its molecular chain contains multiple active sites and can form covalent bonds with phenolic cross-linking agents. P-2 is an acrylamide (AM)-maleic anhydride (MA) binary copolymer with a molecular weight of 4–6 million Daltons, containing only amide groups and anhydride groups (with an anhydride group content of 10%) and fewer reaction sites. P-3 is hydroxypropyl guar gum (HPG), a natural polymer derivative with a molecular weight of 2–3 million Daltons, which forms gels mainly through the interaction between hydroxyl groups and cross-linking agents.

**Figure 2:**
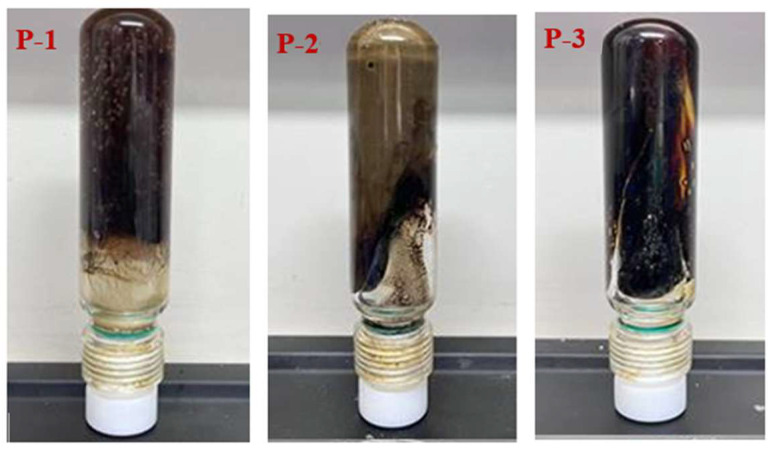
Different types of polymer crosslinking gel morphology.

**Figure 3:**
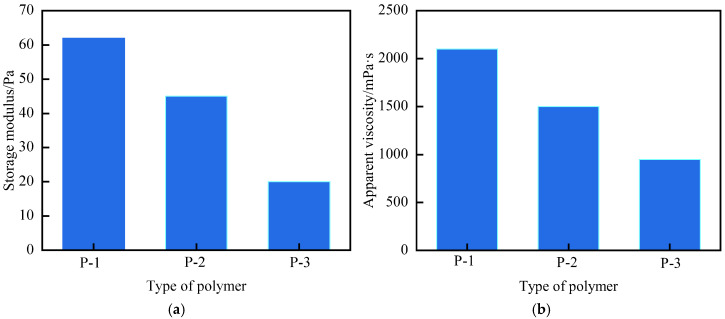
Schematic comparison of temperature resistance for different polymer types: (**a**) Storage Modulus (G′); (**b**) Apparent Viscosity.

The experiment fixed the cross-linking system as follows: 0.5% resorcinol + 0.6% S-Trioxane + 0.8% modified montmorillonite + 14% modified phenolic resin, with 1.0% polymer added and the gelation temperature set at 120 °C. The Sydansk bottle test method and rheometer were used to evaluate the gelation performance. The results are shown in Figure 2 and Figure 3. For P-1, the gel strength reaches grade H, the storage modulus is 62 Pa, the apparent viscosity is 2100 Pa·s, and the gelling time is 6 h (The testing conditions for the storage modulus in the experiment were all set at a frequency of 1 Hz, a strain of 0.1%, and a temperature of 120 °C. The testing conditions for the apparent viscosity were all set at a shear rate of 1 s^−1^ and a temperature of 120 °C). For P-2, the gel strength is grade F (G′ = 35 Pa) with an apparent viscosity of 1500 Pa·s, and the network is loose due to insufficient reaction sites. For P-3, the gel strength at 120 °C is grade D, the storage modulus is 20 Pa, and the apparent viscosity is 950 Pa·s. The excellent performance of P-1 stems from the fact that the strongly polar sulfonic acid groups (-SO_3_^−^) of the AMPS monomer can enhance the hydrophilicity and salt resistance of the molecular chain; the carboxyl groups (-COOH) provided by AA form hydrogen bonds with the hydroxyl groups of the phenolic cross-linking agent, and together with the amide groups (-CONH_2_) of AM, they participate in covalent cross-linking, constructing a dense and elastic network, which provides a basis for thixotropic properties (structural reconstruction after shearing).

**Figure 4:**
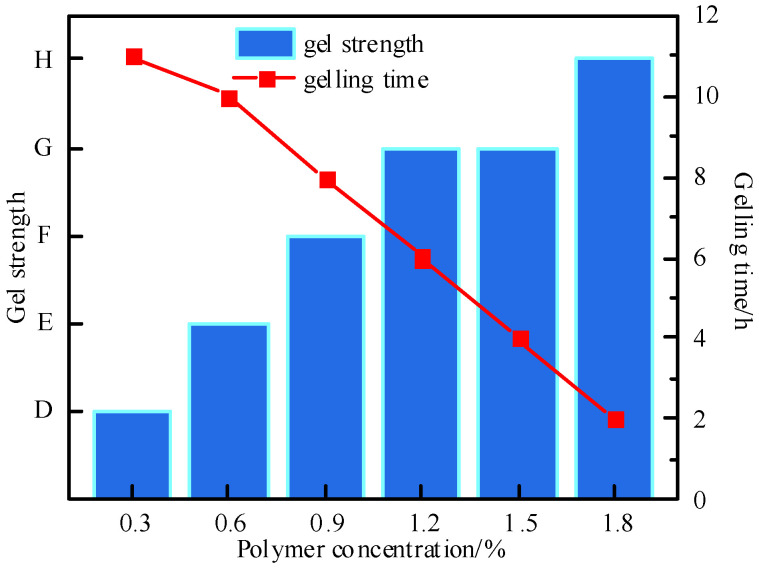
Effect of polymer concentration on the strength of gel formation.

**Figure 5:**
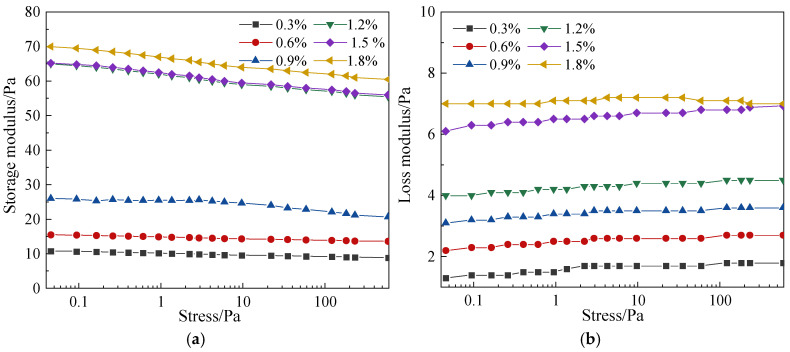
Influence of polymer concentration on the rheological properties of gel systems: (**a**) Storage modulus; (**b**) Loss modulus.

Polymer gel systems with different P-1 concentrations (0.3–1.8%) were prepared to optimize the optimal concentration of the terpolymer P-1. The experimental results are shown in Figure 4 and Figure 5. With the increase in P-1 concentration, the strength of the gel system continuously improved as the polymer concentration increased. The gelling time of the system shortened from 11 h to 2 h, and the storage modulus increased from 12 Pa to 70 Pa. When the concentration was further increased to 1.2%, the gel strength did not increase, indicating that the polymer at a concentration of 1.2% completely reacted with the cross-linking agent. The storage modulus of the gel system was 55 Pa, the loss modulus was 4 Pa, the gel strength of the gel system was the most excellent, and the gel had good integrity and viscoelasticity. Therefore, the optimal concentration of polymer P-1 was selected as 1.2%. Detailed performance data of non-optimal polymers (P2, P3) under different temperatures and concentrations, including gel strength, gelling time, and storage modulus, are provided in Table 1 to avoid cluttering the main text.

#### 3.1.2. Optimization of Crosslinker Type and Concentration

Cross-linking agents form a three-dimensional network by reacting with functional groups such as amide groups and carboxyl groups of the terpolymer (AM-AA-AMPS). Their types and concentrations directly affect the gelation rate, strength, and thermal stability of the gel. Considering the characteristics of the functional groups of the polymer in the system, aldehyde and phenolic cross-linking agents were screened, respectively, and then the optimal combination was determined through concentration optimization [40]. The experiment fixed the terpolymer concentration at 1.2%, the flow pattern regulator at 0.8%, and the modified phenolic resin at 14%. The performance was evaluated by gelling time, storage modulus, and aging stability at 120 °C.

##### Optimization of Aldehyde Crosslinker

The synergistic effects of three aldehyde cross-linking agents (formaldehyde, S-Trioxane, and hexamethylenetetramine (HMTA)) with hydroquinone at different addition amounts were compared. The experimental results are shown in Table 2. Formaldehyde achieved gelation the fastest, taking about 1.5 h. This is mainly because formaldehyde is quickly released and directly participates in the cross-linking reaction, resulting in a very fast cross-linking reaction rate and a rapid increase in gel strength grade. However, after aging at 120 °C for 7 days, the polymer dehydrated severely and began to degrade. S-Trioxane slowly releases formaldehyde at high temperatures, and its reaction rate with phenols matches the diffusion of polymer chains. At a concentration of 0.6%, the gelling time is 6 h, and the dehydration rate after 7 days of aging is only 8%. When the concentration of HMTA is 0.6%, the gelling time is 8 h, and the dehydration rate after 7 days of aging is 12%. In addition, there is network repulsion between HMTA and modified phenolic resin. Therefore, S-Trioxane was selected as the aldehyde cross-linking agent for the gel system.

**Figure 6:**
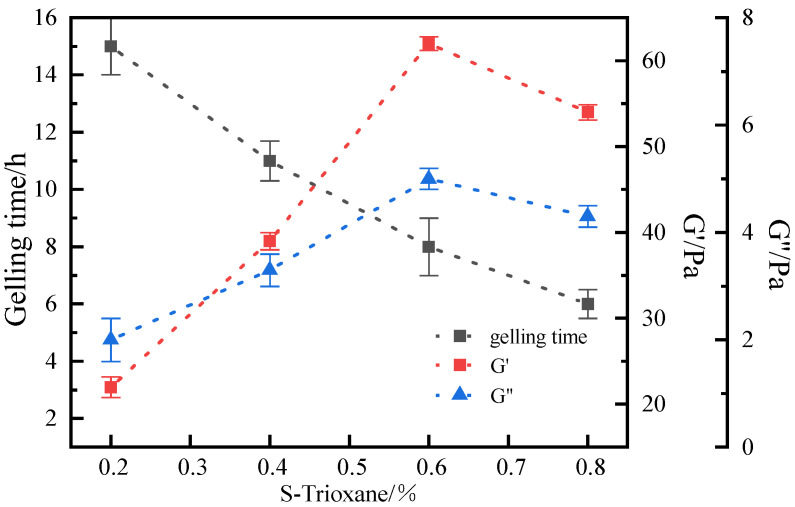
Influence of S-Trioxane Concentration on Gelling time and Storage Modulus of Gel System.

The optimal concentration (0.2–0.8%) of S-Trioxane in the gel system was selected under the high temperature condition of 120 °C. The experimental results showed (Figure 6) that polymer gel systems with obvious strength could be formed within the set concentration range of S-Trioxane, and the gelling time was in the range of 6–15 h. With the increase in cross-linking agent concentration, the gelling time of the system decreased. Within the concentration range of 0.3–0.6%, the strength of the system increased significantly with the increase in S-Trioxane concentration. When the concentration of S-Trioxane was 0.6%, the gel strength reached grade H, and the storage modulus and loss modulus of the gel system reached the maximum values of 62 Pa and 5 Pa, respectively, indicating that the gel system exhibited excellent viscoelastic properties. When the concentration exceeded 0.6%, the storage modulus and loss modulus of the gel system decreased instead. The reason is that when the cross-linking agent exceeds a certain concentration, the gel system undergoes excessive cross-linking, and the cross-linking reaction rate is too fast, making it difficult to form a stable cross-linking structure. Considering factors such as gel strength and gelling time comprehensively, the optimal concentration of S-Trioxane was selected as 0.6%.

##### Optimization of Phenolic Crosslinker

Using terpolymer P-1 (with a concentration range of 0.3–1.5%) as the main agent of the gel, the same concentrations of 0.6% S-Trioxane and 0.5% phenolic cross-linking agents were added to compare the effects of three phenolic cross-linking agents (phenol, catechol, and hydroquinone) on the gelation performance of the gel system under high-temperature conditions [25]. The experimental results are shown in Table 3. When phenol was used as the cross-linking agent, the gel strength formed by the gel system was too weak. Due to the low activity of the hydroxyl groups in phenol, the reaction with aldehydes was incomplete, and the network collapsed after aging. The system using hydroquinone as the phenolic cross-linking agent showed good gelation effect, with a gelling time of 4–11 h and a gel strength reaching grade D or above. The dehydration rate of the gel system after 7 days of aging was less than 15%, indicating excellent long-term stability. This is because the para-hydroxyl groups of hydroquinone have no steric hindrance and can fully react with aldehydes. When catechol was used as the cross-linking agent, the gel system had a relatively good gelation effect, with a gelling time of 8–18 h and a gel strength reaching grade D or above. However, the dehydration rate of the gel system after 7 days of aging was more than 15%, showing poor long-term stability. The reason is that the ortho-hydroxyl groups of catechol have steric hindrance, resulting in insufficient cross-linking density. Therefore, hydroquinone was selected as the optimal choice, as its reaction activity and cross-linking efficiency are more suitable for the requirements of the system.

**Figure 7:**
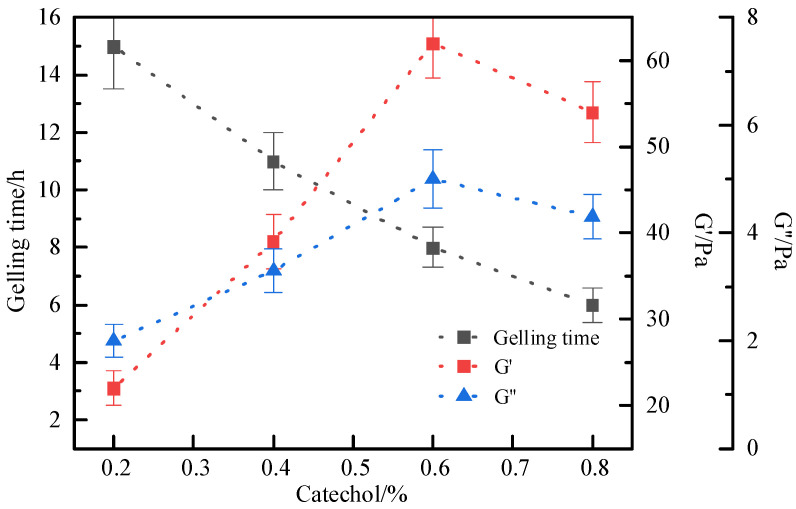
Effect of catechol concentration on gelling time and storage modulus.

The influence law of hydroquinone concentration on the gelation of the gel system was further investigated. The results are shown in Figure 7. With the increase in hydroquinone concentration, the gelling time of the system is shortened, and the gel strength first increases and then decreases. When the concentration of hydroquinone is 0.5%, the gel strength reaches the highest value, with the storage modulus and loss modulus being 65 Pa and 9 Pa, respectively. However, when the concentration of the hydroquinone cross-linking agent continues to increase beyond 0.5%, the complex modulus of the gel system decreases instead, and the gel strength weakens. This is consistent with the phenomenon observed in the optimization experiment of aldehyde cross-linking agent concentration. Excessively high cross-linking agent concentration leads to excessive cross-linking in the gel system, which will result in the degradation of gel performance. Therefore, the optimal concentration of hydroquinone is 0.5%. In summary, the optimal formula of the cross-linking system is 0.5% hydroquinone + 0.6% S-Trioxane.

#### 3.1.3. Optimization of Flow Pattern Regulators Type and Concentration

**Figure 8:**
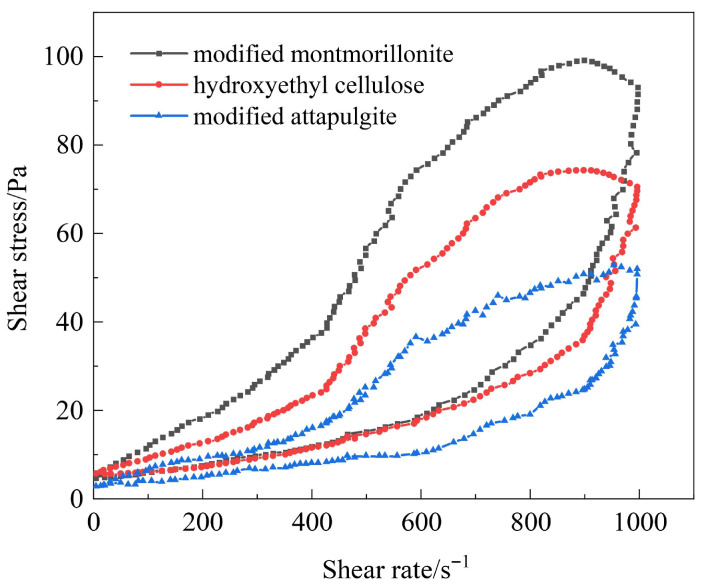
Cyclic test diagram of gel solutions with different types of flow pattern regulators.

Flow pattern regulators control the thixotropic properties of gels through physical actions [41]. They need to be compatible with the amide groups, carboxyl groups, and sulfonic acid groups of the terpolymer (AM-AA-AMPS) without interfering with covalent cross-linking and the reinforcing effect of the resin network. The screening results of three materials, namely modified attapulgite, hydroxyethyl cellulose, and modified montmorillonite, based on the characteristics of functional groups, are shown in Figure 8. At the same shear rate, the shear stress of modified montmorillonite is the highest, followed by hydroxyethyl cellulose, and modified attapulgite is the lowest, indicating that modified montmorillonite can significantly enhance gel strength and stability. Modified attapulgite enhances shear thinning through electrostatic adsorption. After gel formation at 120 °C, the cyclic shear hysteresis loop area is 85 Pa·s, the thixotropic recovery rate is 75%, and the stability is poor. Hydroxyethyl cellulose thickens through physical entanglement, with a hysteresis loop area of 105 Pa·s and a recovery rate of 82%. The lamellar structure of modified montmorillonite stably adsorbs with polar functional groups, with a hysteresis loop area of 118 Pa·s and a recovery rate of 90%. At high temperatures, the lamellae fix cross-linking points and cooperate with the resin network to maintain thixotropic stability. Based on comprehensive performance, modified montmorillonite is selected as the flow pattern regulator for the thixotropic polymer gel system.

**Figure 9:**
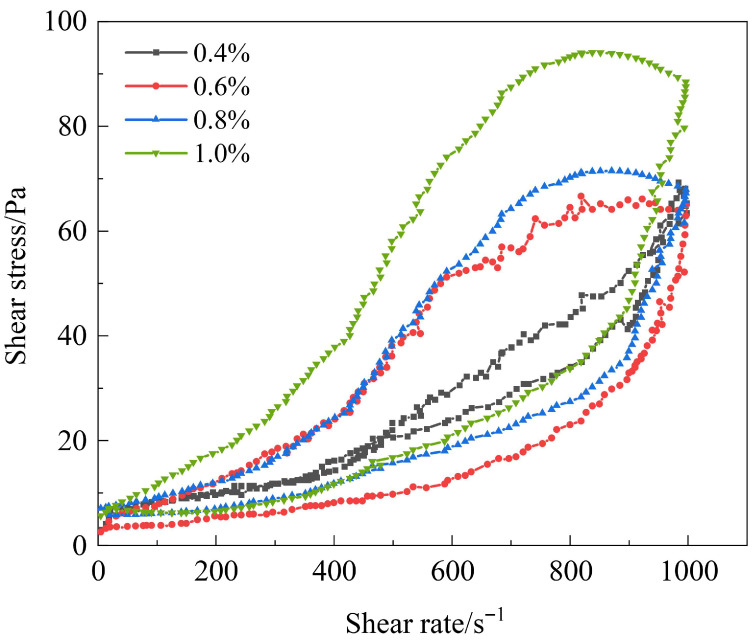
Cyclic test diagram of gel solutions with different concentrations of modified montmorillonite.

The influence of modified montmorillonite concentration on the rheological properties of the gel system was further investigated. With other components fixed as 1.2% AM-AA-AMPS + 0.5% hydroquinone + 0.6% S-Trioxane + 14% resin, the concentration of montmorillonite was adjusted (0.4–1.0%) to test its influence on the gelation of the gel system. It can be seen from the optimization (Figure 9) of modified montmorillonite concentration that as the concentration of modified montmorillonite increases, the shear stress of the gel system first increases and then decreases, reaching the maximum value at 0.8%, indicating that the gel strength is optimal at this time. When the concentration of modified montmorillonite is 0.4%, the hysteresis loop area is small and the thixotropic recovery rate is low. Due to the insufficient content of modified montmorillonite, the reinforcing effect is not fully exerted. When the concentration increases to 0.6%, the hysteresis loop area gradually increases and the recovery rate improves, indicating that the interaction between modified montmorillonite, AM-AA-AMPS, and modified phenolic resin is enhanced, and the three-dimensional network structure is more stable. When the concentration is 0.8%, the hysteresis loop area is the largest, the thixotropic recovery rate is the highest, and the thixotropic performance is the best. At this concentration, an appropriate amount of modified montmorillonite effectively interacts with the sulfonic acid groups of AM-AA-AMPS and the hydroxyl groups of modified phenolic resin, forming a stable composite network, which strengthens the thixotropic properties and stability of the gel. When the concentration reaches 1.0%, both the shear stress and the hysteresis loop area decrease, and the recovery rate decreases slightly. This is because excessively modified montmorillonite leads to excessively strong intermolecular interactions, which destroy the original network structure and affect the gel performance. Therefore, 0.8% modified montmorillonite is selected. Through physical adsorption, it forms a stable “cross-linking point—lamella” structure with the terpolymer, and cooperates with the network-reinforcing effect of the resin curing agent to improve the thixotropic properties and high-temperature shear resistance of the gel.

#### 3.1.4. Optimization of Resin Hardener Type and Concentration

To enhance the long-term stability of the thixotropic polymer gel system in the high-temperature environment of the formation [42], this study introduces a resin curing agent—modified phenolic resin into the construction of the thixotropic polymer gel system. The modified phenolic resin molecules are rich in highly reactive functional groups, such as -CH_2_OH (hydroxymethyl), -NH- (imino), -NH_2_ (amino), etc. Various polycondensation reactions can occur between these functional groups. For example, dehydration condensation reaction takes place between hydroxymethyl groups to form ether bonds -CH_2_-O-CH_2_-; dehydration condensation reaction can also occur between amino groups and hydroxymethyl groups, forming methylene bridge bonds -CH_2_-NH- after removing one molecule of water. Through these reactions, the modified phenolic resin molecules are continuously connected and intertwined with each other, gradually forming a three-dimensional network structure. This three-dimensional network structure, like a solid framework, namely the resin network framework, can significantly enhance the gel-forming strength and viscoelasticity of the gel system.

In a high-temperature environment, the three-dimensional network structure formed by the modified phenolic resin can effectively inhibit the thermal movement and degradation of molecular chains in the gel system, thereby greatly improving the temperature resistance and long-term stability of the gel. This is of great significance for improving the high-pressure bearing plugging capacity of the gel plugging agent for large fracture leakage channels after high-temperature gelation, enabling the new system to maintain a stable plugging effect in the high-temperature and high-pressure formation environment.

**Figure 10:**
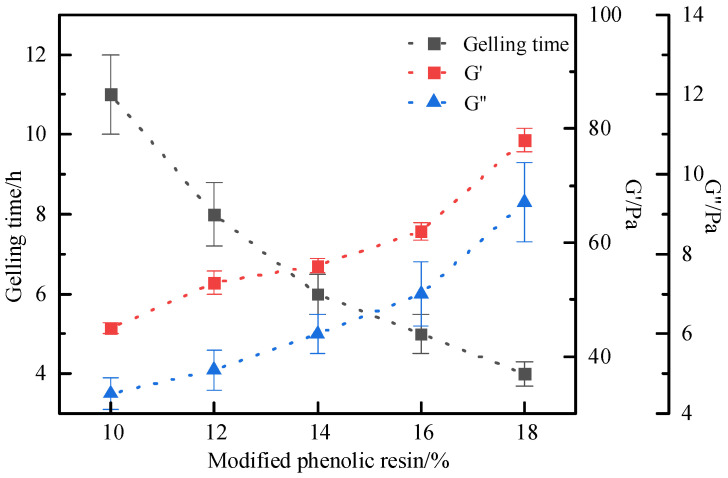
Effect of modified phenolic resin concentration on gelling time and storage modulus.

The influence of modified phenolic resin concentration on the gelation performance of the gel system was further investigated. The concentrations of other components were fixed as 1.2% AM-AA-AMPS + 0.5% hydroquinone + 0.6% S-Trioxane + 0.8% modified montmorillonite, while the concentration of modified phenolic resin was adjusted. The results are shown in Figure 10. At 120 °C, the modified phenolic resin within the concentration range of 10–18% can form a complete gel with other components in the system. The concentration of modified phenolic resin has a significant impact on the strength and gelling time of the gel system. As the resin concentration increases, the gelling time of the system shortens from 11 h to 4 h, and the gel strength increases continuously. When the resin concentration is 14%, the storage modulus and loss modulus reach 56.4 Pa and 6.2 Pa, respectively, and the strength can meet the requirements of zonal isolation. Excessively high resin concentration will damage the viscoelasticity of the gel, resulting in obvious dehydration of the gel, reduced toughness of the gel, and a relatively short gelling time, which makes it difficult to control the curing process. To sum up, the optimal concentration of modified phenolic resin is 14%. Finally, the final formula of the thixotropic polymer gel system is determined as follows: 1.2% AM-AA-AMPS + 0.5% hydroquinone + 0.6% S-Trioxane + 0.8% modified montmorillonite + 14% modified phenolic resin.

Beyond laboratory performance, the practical viability of the gel system is underscored by its industrial compatibility and cost-effectiveness. All components are commercially available industrial-grade chemicals, ensuring a reliable supply chain for scale-up. The synthesis process, involving simple aqueous-phase mixing and thermal activation, aligns with standard oilfield chemical preparation protocols and requires no specialized equipment. An economic analysis based on current material costs estimates the price of the optimized formulation at approximately 8500 CNY per ton. This positions the system favorably within the market: it is significantly more economical than high-performance epoxy resin systems (25,000–42,000 CNY/ton) and only marginally more expensive than conventional chromium-crosslinked gels (~7800 CNY/ton). This modest cost premium is justified by the system’s exceptional high-temperature stability, elimination of environmentally hazardous heavy metals, and superior plugging reliability, presenting a compelling value proposition for demanding high-temperature wellbore sealing applications.

### 3.2. Molecular Structure Characterization of the Polymer Gel System

**Figure 11:**
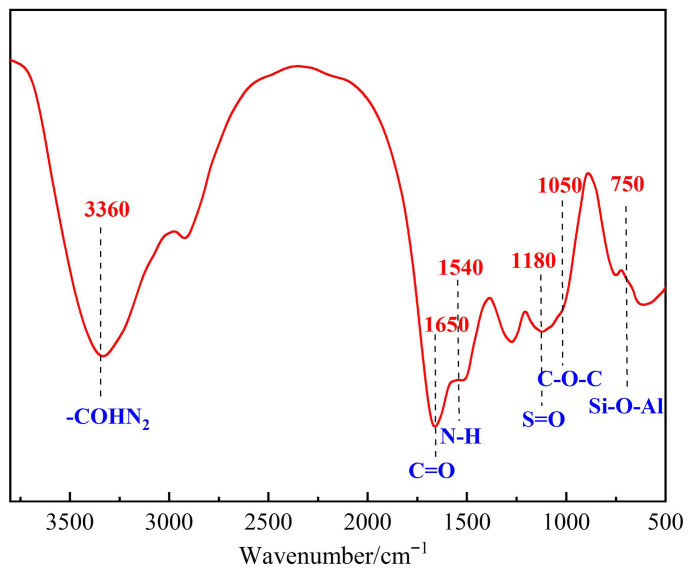
FTIR structural characterization of the polymer gel system.

The dried gel samples were analyzed using a Fourier transform infrared spectrometer (scanning range: 400~4000 cm^−1^), and the results are shown in Figure 11. The strong absorption peak at 3360 cm^−1^ corresponds to the N-H stretching vibration of the amide group (-CONH_2_). The hydrogen bonding interaction with the hydroxyl group (-OH) in the modified phenolic resin broadens the peak shape. The peak at 1650 cm^−1^ is the amide I band (C=O stretching vibration), and the peak at 1540 cm^−1^ is the amide II band (N-H bending vibration). Compared with the pure terpolymer, the intensity ratio of the two peaks (1650/1540) increases from 1.2 to 1.5, indicating that the cross-linking agent (catechol-HMTA) forms covalent bonds with the polymer, enhancing the ordered arrangement of amide groups. The S=O stretching vibration peak of the sulfonic acid group (-SO_3_^−^) appears at 1180 cm^−1^, and the C-O-C ether bond vibration peak in the phenolic resin is observed at 1050 cm^−1^, which proves that the sulfonic acid groups of the terpolymer are successfully combined with the resin network. The Si-O-Al vibration peak of modified montmorillonite at 750 cm^−1^ indicates that its layered structure stably exists in the gel. The above characteristic peaks confirm the chemical cross-linking between the terpolymer and the cross-linking agent, as well as the resin curing agent, and the physical doping of the flow pattern regulator, forming a composite structure with the synergistic effect of “covalent bonds—hydrogen bonds—physical adsorption”.

**Figure 12:**
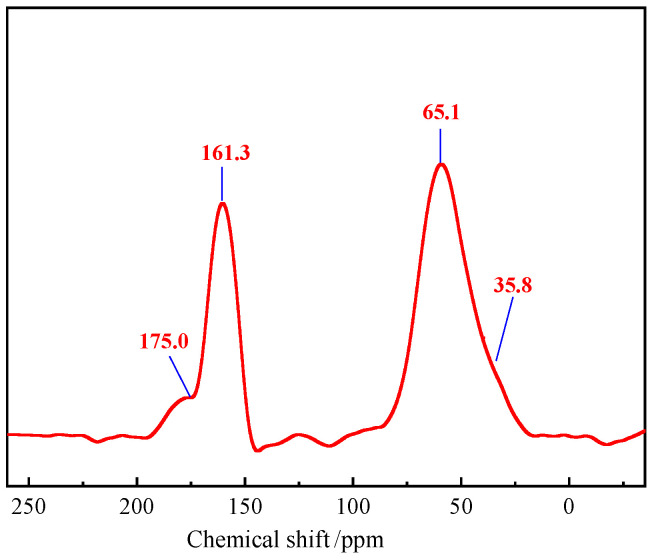
NMR analysis of the polymer gel system.

A Bruker AVANCE 400 MHz nuclear magnetic resonance spectrometer was used to perform ^13^C NMR testing on dried gel samples with deuterated dimethyl sulfoxide (DMSO-d_6_) as the solvent, so as to characterize the interactions between polymer chains, cross-linking agents, and flow pattern regulators. The ^13^C-NMR spectrum of the thixotropic polymer gel system is shown in Figure 3, Figure 4, Figure 5, Figure 6, Figure 7, Figure 8, Figure 9, Figure 10, Figure 11 and Figure 12. The peak at δ = 175.0 corresponds to the C=O bond in the amide group of the terpolymer. The formaldehyde released by S-Trioxane forms a C-N covalent bond with the amide group (-CONH_2_), which reduces the electron cloud density and enhances the deshielding effect, confirming the formation of covalent cross-linking. The peak at δ = 161.3 is attributed to the benzene ring carbon atoms of hydroquinone and modified phenolic resin. The peak at δ = 65.1 corresponds to the methylene carbon (-CH_2_-) produced by the decomposition of S-Trioxane, which combines with the nitrogen atom of the amide group (-CONH-CH_2_-) to form a stable cross-linking bridge bond. The peak intensity is 29% lower than that of pure S-Trioxane (δ = 92.0), indicating that it participates in the reaction and is embedded in the gel network. The peak at δ = 35.8 comes from the methyl carbon (-CH_3_) in the AMPS unit. Due to the strong electron-withdrawing effect of the sulfonic acid group (-SO_3_^−^), the chemical shift is stable, confirming that it does not participate in cross-linking and only assists in network stabilization through electrostatic interaction. The system forms a composite cross-linked structure with both rigidity and stability through the covalent cross-linking between amide groups and S-Trioxane, the conjugation between hydroquinone and benzene rings, and the interpenetrating network of phenolic resin.

### 3.3. Evaluation of Performance of Thixotropic Polymer Gel System

#### 3.3.1. Shear Resistance of Polymer Gel System

**Figure 13:**
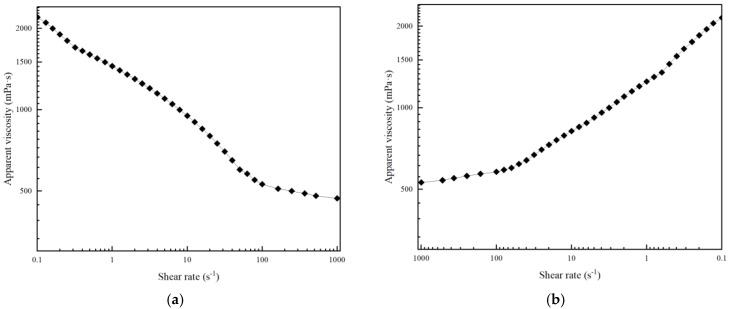
Apparent viscosity versus shear rate at 120 °C: (**a**) Forward shear; (**b**) Reverse shear.

Shear resistance is a key indicator to measure the structural stability of the gel during pumping and formation flow [43]. A rotational rheometer was used to conduct dynamic shear tests on the thixotropic polymer gel system at 120 °C, so as to evaluate its viscosity retention capacity and structural recovery characteristics under different shear rates. The test results are shown in Figure 13. Within the shear rate range of 0.1~1000 s^−1^, the apparent viscosity of the gel solution decreases from 2200 mPa·s to 480 mPa·s, showing significant shear thinning characteristics. This is due to the oriented arrangement of modified montmorillonite lamellae under shear and the disentanglement of terpolymer molecular chains, ensuring that the system can be smoothly injected through pumping equipment. When the shear rate is restored to 0.1 s^−1^, the viscosity rises back to 2150 mPa·s, with a recovery rate of 97.7%, indicating that after the shear stops, the electrostatic adsorption and molecular entanglement between montmorillonite lamellae and polymer chains can quickly rebuild the network structure. Through the synergistic effect of “covalent cross-linking—physical adsorption—network interpenetration”, the optimized formula exhibits excellent shear resistance stability, which can meet the requirements of plugging operations under high shear conditions in complex formations.

The pumpable-plugging transition analysis above is based on apparent viscosity and storage modulus data, with inherent limitations due to unmeasured yield stress and uncorrected wall slip. For example, the actual yield stress of the gel may affect the minimum shear rate required for flow, which could slightly adjust the predicted downhole pumping parameters. Future yield stress measurements (via stress-ramp tests) will provide a more accurate basis for optimizing pumping conditions. Combined with the rheological data in Figure 13 and the gel strength characteristics, the transition mechanism of the gel system from pumpable to plugging state under downhole conditions can be clarified. During wellbore pumping, the shear rate in the drill pipe annulus typically ranges from 100 to 1000 s^−1^; within this range, the gel’s apparent viscosity decreases from 2200 mPa·s to 480 mPa·s (Figure 13a), showing significant shear thinning that ensures smooth transportation without excessive friction loss, meeting pumpability requirements. When pumping stops and the gel enters lost circulation channels, the shear rate drops to near 0.1 s^−1^ (downhole static environment); the gel’s apparent viscosity rapidly recovers to 2150 mPa·s (recovery rate 97.7%, Figure 13b), with the storage modulus maintaining 62 Pa (Section 3.1.1) and gel strength reaching grade H. Relying on the synergistic effect of “covalent cross-linking—hydrogen bonding—physical adsorption” (Section 3.2, the gel reconstructs a three-dimensional network, forming an elastic plugging layer that resists formation pressure.

#### 3.3.2. Thixotropic Behavior of Polymer Gel System

**Figure 14:**
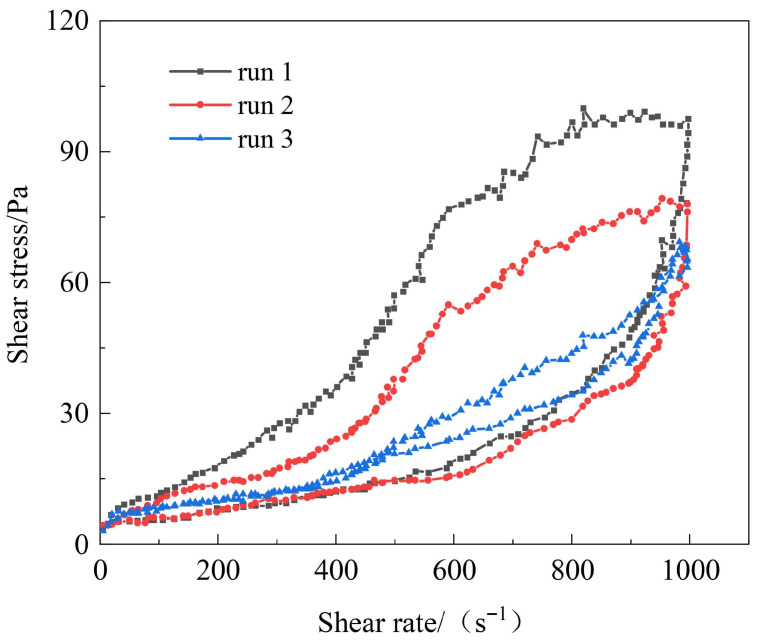
Hysteresis regions during cyclic shear testing.

Thixotropic property is a core index for evaluating the reversible structural reconstruction ability of a gel system during shear-stand cycles. A rotational rheometer was employed to perform cyclic shear tests (0.1~1000~0.1 s^−1^) on the thixotropic polymer gel system at 120 °C, with the results presented in Figure 14. In the first cycle, the area of the hysteresis loop was the largest. This is because, in the initial state, the molecular chains of the terpolymer are entangled with each other, and the modified montmorillonite lamellae form a physical adsorption network. During shearing, the chain entanglements are untangled and the lamellae are oriented, while there is a lag in network reconstruction during the recovery phase. In the subsequent cycles, the characteristics of the thixotropic loops are similar, but the hysteresis area gradually decreases. This is attributed to the repeated shearing, which causes excessive stretching of some molecular chains or irreversible bond breakage (local breakage of amide bonds). Meanwhile, the active sites on the montmorillonite lamellae are saturated with adsorption, leading to a weakened network reconstruction ability, and it is difficult for the molecular chains to fully recover to their initial entangled state. After three cycles, the ascending and descending curves nearly overlap. The stability of thixotropic recovery in cyclic shear is coupled with the gel’s elastic structural characteristics, indirectly reflecting the correlation between thixotropy and yield stress trends. In the first cycle, the largest hysteresis loop area corresponds to the initial entanglement of terpolymer chains and physical adsorption of modified montmorillonite; during shearing, the network is temporarily destroyed, and the recovery lag indicates time-dependent cross-linking point reconstruction, while the storage modulus remains >55 Pa after shearing, suggesting the elastic skeleton is not completely broken. In subsequent cycles, the gradual reduction in hysteresis loop area is due to partial adsorption site saturation, but the overlapping of ascending/descending curves after three cycles indicates a stable “shear-destruction-reconstruction” process. With the storage modulus retention rate >80% after cyclic shearing, the gel’s elastic structural strength is maintained, ensuring thixotropic recovery does not cause irreversible plugging capacity loss.

The evolution of the hysteresis loop area over successive shear cycles (Figure 14) offers valuable insights into the structural robustness of the gel. The reduction in hysteresis from the first to later cycles indicates a minor, irreversible reorganization within the network, potentially due to the saturation of highly active adsorption sites on the montmorillonite or the irreversible sliding of polymer chains. Crucially, the subsequent stabilization of the hysteresis loop, with the ascending and descending curves nearly overlapping after three cycles, demonstrates that the gel reaches a steady, resilient state. This stable thixotropic performance, corroborated by the high retention of storage modulus after prolonged aging (Section 3.3.4), confirms that the elastic backbone—comprised of covalent cross-links and the resin network—remains largely undisturbed. This ensures that the gel’s rapid recovery capability does not compromise its long-term mechanical integrity and sealing function.

#### 3.3.3. High-Temperature Gelation Performance

**Figure 15:**
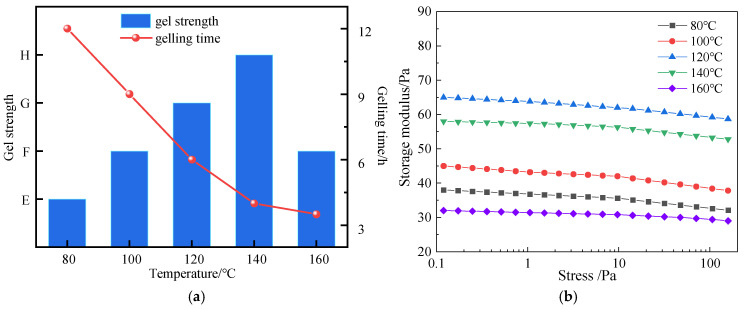
Influence of different temperatures on the gelation performance of the gel system: (**a**) Gelation effect; (**b**) Storage modulus.

High-temperature gelation performance is a key indicator for evaluating whether a gel can form a stable plugging structure in a reservoir environment. The Sydansk bottle test method combined with rheometer testing was used to evaluate the gelling time, strength, and stability of the thixotropic polymer gel system within the temperature range of 80~160 °C. The results show (as shown in Figure 15) that at 80 °C, the gelling time is 12 h, the gel strength reaches grade E, and the storage modulus is 38 Pa; at 120 °C, the gelling time is shortened to 6 h, and G′ rises to 65 Pa. This is because high temperature accelerates the cross-linking reaction between hydroquinone and S-Trioxane, and the rigid network of modified phenolic resin cooperates with montmorillonite lamellae to enhance structural compactness; at 160 °C, the gelling time is further shortened to 3.5 h, G′ remains at 32 Pa, the gel strength still reaches grade F, and there is no dehydration or cracking. The lamellar structure of modified montmorillonite effectively inhibits the thermal degradation of the cross-linked network at high temperatures, and the sulfonic acid groups (-SO_3_^−^) of the terpolymer stabilize the adsorption with montmorillonite through strong polarity, ensuring gelation stability in high-temperature environments and meeting the plugging needs of medium and high-temperature reservoirs.

#### 3.3.4. High-Temperature Stability of Polymer Gel System

**Figure 16:**
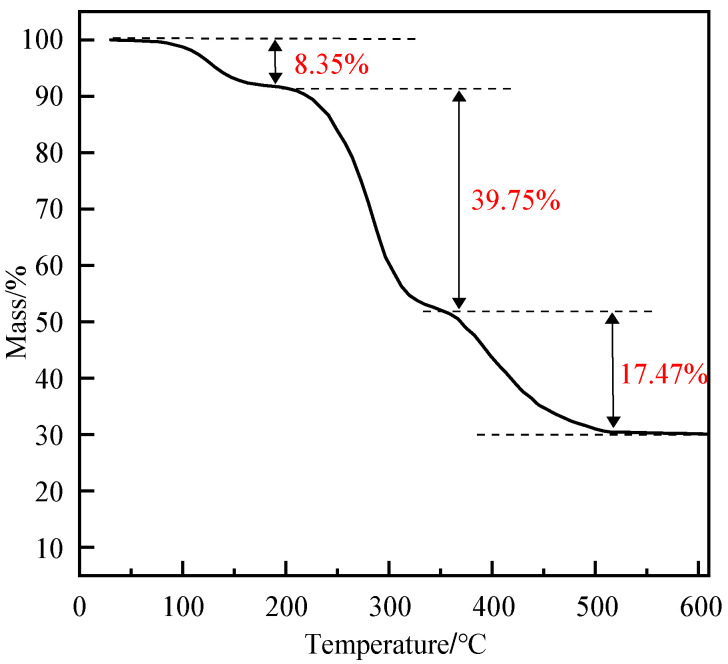
TGA curve of polymer gel material.

To evaluate the thermal stability of the polymer gel system, a thermogravimetric analyzer was used to conduct thermal stability tests on the gel system [44]. Under a nitrogen atmosphere (with a flow rate of 20 mL/min), the temperature was raised from 30 °C to 600 °C at a rate of 10 °C/min. The thermogravimetric curve (Figure 16) shows that in the first weight loss stage (50~200 °C), the weight loss rate is approximately 8.35%, which corresponds to the evaporation of free water and part of the bound water in the gel. In the second weight loss stage (200~350 °C), the weight loss rate is about 39.75%, mainly resulting from the decomposition of amide groups and carboxyl groups in the polymer chains as well as the thermal decomposition of phenolic resin. In the third weight loss stage (350~500 °C), the weight loss rate is 17.47%, corresponding to the breakage of the main chain skeleton. The residual mass is approximately 30%, which consists of montmorillonite and inorganic residues, further verifying its high-temperature stability.

**Figure 17:**
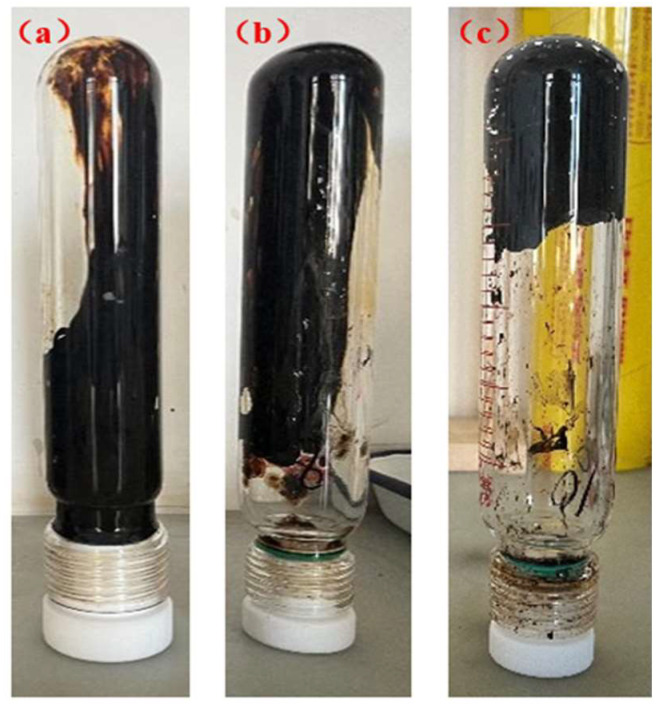
Gelation state of thixotropic gel system after aging for 7 days at different temperatures: (**a**) 120 °C; (**b**) 130 °C; (**c**) 140 °C.

**Figure 18:**
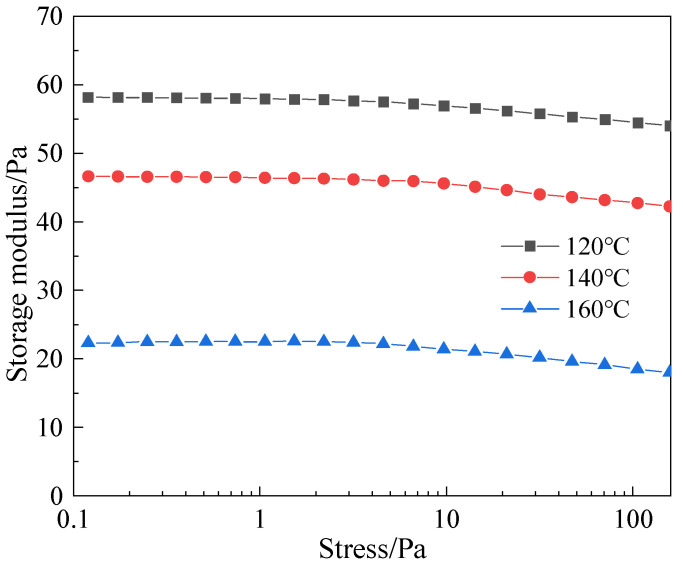
Influence of 7-day aging of thixotropic gel system on storage modulus at different temperatures.

High-temperature stability is a core indicator for measuring the ability of a gel to maintain structural integrity in a long-term high-temperature reservoir environment. The stability of the thixotropic polymer gel system at 120~160 °C was evaluated through high-temperature aging experiments. The results show (as shown in Figure 17 and Figure 18) that after aging at 120 °C for 7 days, the gel showed no obvious dehydration or cracking, the mass loss rate was only 5.2%, and the storage modulus decreased from the initial 65 Pa to 58 Pa (retention rate of about 90.0%); after aging at 140 °C for 7 days, the mass loss rate increased to 8.5%, and G′ was 46 Pa (retention rate of about 80.0%), still maintaining grade H strength. After aging at 160 °C for 7 days, the mass loss rate was 12.3%, G′ decreased to 22 Pa (retention rate of about 70.0%), and the strength dropped to grade E, but there was no collapse phenomenon. These indicate that the thixotropic polymer gel system has excellent thermal stability at high temperatures.

#### 3.3.5. Plugging Performance of Polymer Gel System

Plugging performance is a key indicator for measuring the effectiveness of thixotropic polymer gel systems in practical applications, and it is crucial for the effective treatment of lost circulation formations. In this study, a sand-packed tube model was used to simulate the porous media of the formation, and the plugging performance of the thixotropic polymer gel system was tested and analyzed. It can be seen from the data in Table 4 that after the gel system was aged at 120 °C for 1 day, the 100 m equivalent pressure-bearing capacity under water flooding and gas flooding conditions was 24.0 MPa and 17.0 MPa, respectively. After aging for 3 days, the 100 m equivalent pressure-bearing capacity decreased to 22.0 MPa and 15.0 MPa, respectively. When the temperature increased to 140 °C, the pressure-bearing capacity of the gel system decreased to a certain extent. After aging at 140 °C for 1 day, the 100 m equivalent pressure-bearing capacity under water flooding and gas flooding conditions was 21.0 MPa and 14.0 MPa, respectively, and further decreased to 19.0 MPa and 12.0 MPa after aging for 3 days. It can be seen that the plugging capacity of the gel system decreases with the increase in temperature or aging time.

When water source and nitrogen source are used as displacement media, respectively, due to the differences in physical properties between water and nitrogen, the breakthrough pressure of the gel system under the action of different media is also different. By comparing the breakthrough pressure when water and nitrogen are used as displacement media, it is found that the breakthrough pressure of the gel system for nitrogen is significantly lower than that for water. This is because gas molecules are more likely to penetrate into the microscopic pores of the gel than water molecules, resulting in increased plugging difficulty. Therefore, the gel system has a better plugging effect on water.

The reason why the thixotropic polymer gel system can exhibit good plugging performance is mainly attributed to the cross-linked network formed by the AM-AA-AMPS terpolymer with hydroquinone and S-Trioxane, which provides a basic plugging framework, and the addition of modified montmorillonite further enhances the strength and stability of the gel. In addition, the modified phenolic resin plays an important supporting role in high-temperature environments, collectively ensuring the plugging effect of the new system under different conditions.

## 4. Conclusions

(1) In this study, through the screening of core components and concentration optimization, the optimal formula of the new thixotropic polymer gel system was obtained as follows: 1.2% AM-AA-AMPS terpolymer, 0.5% hydroquinone, 0.6% S-Trioxane, 0.8% modified montmorillonite, and 14% modified phenolic resin. This formula has a gelling time of 6 h, a strength reaching grade H, and a storage modulus of 62 Pa, exhibiting good initial structure and viscoelasticity.

(2) The gel system has excellent comprehensive performance: it shows significant shear thinning characteristics with a viscosity recovery rate of 97.7% after shearing, and the thixotropic recovery is stable; it forms a complete gel at a high temperature of 160 °C, with a dehydration rate of only 8.5% after aging at 140 °C for 7 days, demonstrating outstanding thermal stability; the converted pressure bearing capacity per 100 m under water flooding at 120 °C is 24.0 MPa, indicating reliable plugging efficiency.

(3) Structural characterization shows that the system forms a composite structure with the synergistic effect of “covalent bond—hydrogen bond—physical adsorption” through the covalent cross-linking between the terpolymer and cross-linking agent, the physical adsorption of modified montmorillonite, and the network reinforcement of modified phenolic resin. This is the core mechanism for its high performance, providing material and theoretical support for wellbore plugging in complex environments.

(4) All components are commercially available oilfield chemicals with an estimated formulation cost of ~8500 CNY/ton. Pilot trials in two Xinjiang Oilfield wells (BHT > 145 °C) are scheduled for Q1 2026. The complete absence of regulated heavy metals constitutes a significant environmental advantage. Comprehensive environmental fate assessment and the advanced rheological protocols described in Section 3.3 will be completed prior to full-scale deployment.

## Figures and Tables

**Table 1:** Performance Comparison of P1, P2, and P3 Polymers Under Different Concentrations (120 °C, 7-Day Aging).

Polymer Type	Polymer Concentration (%)	Gel Strength	Gelling Time (h)	Dehydration Rate After 7-Day Aging (%)	Storage Modulus (G′, Pa)
P1	0.3	D	11.0	14.2	12.3
	0.6	E	10.2	12.5	25.6
	0.9	F	8.1	9.8	42.1
	1.2	H	6.0	8.5	62.0
	1.5	H	4.3	8.2	68.5
	1.8	H	2.1	10.3	65.2
P2	0.3	-	-	-	-
	0.6	C	15.2	22.3	8.5
	0.9	D	12.5	19.8	18.7
	1.2	F	9.8	16.5	35.0
	1.5	F	7.2	15.1	38.2
	1.8	G	5.1	14.8	42.3
P3	0.3	-	-	-	-
	0.6	-	-	-	5.2
	0.9	D	18.5	28.6	12.8
	1.2	D	15.3	25.4	20.0
	1.5	E	12.1	23.7	25.6

**Table 2:** Effect of different aldehydes crosslinking agents on gelling time and thermal stability.

Aldehyde Crosslinker and Concentration (%)		Hydroquinone Concentration (%)	Gelling Time (h)	Gelling Effect and Thermal Stability
Formaldehyde	0.3	0.5	2.5	Gel strength grade E, degraded at high temperature after 7 d
	0.6		2.0	Gel strength grade F, degraded at high temperature after 7 d
	0.9		1.5	Gel strength grade F, dehydration rate 20% after 7 d
S-Trioxane	0.3	0.5	8.0	Gel strength grade F, dehydration rate 12% after 7 d
	0.6		6.0	Gel strength grade F, dehydration rate 12% after 7 d
	0.9		4.5	Gel strength grade H, dehydration rate 10% after 7 d
HMTA	0.3	0.5	9.0	Gel strength grade G, dehydration rate 15% after 7 d
	0.6		8.0	Gel strength grade H, dehydration rate 12% after 7 d
	0.9		6.5	Gel strength grade H, dehydration rate 14% after 7 d

**Table 3:** Effect of Different Phenolic Crosslinkers on Gel System Performance.

Crosslinker and Concentration (%)	Polymer Concentration (%)	Gelling Time (h)	Gelation Quality and Stability
0.5% Phenol + 0.6% S-Trioxane	0.3	-	Strength too weak
	0.6	-	
	0.9	-	
	1.2	-	
	1.5	-	
0.5% Hydroquinone + 0.6% S-Trioxane	0.3	11	Gel strength grade D, dehydration < 15% after 7 d
	0.6	10	Gel strength grade E, dehydration < 15% after 7 d
	0.9	8	Gel strength grade F, dehydration < 15% after 7 d
	1.2	6	Gel strength grade G, dehydration < 10% after 7 d
	1.5	4	Gel strength grade G, dehydration < 10% after 7 d
0.5% Catechol + 0.6% S-Trioxane	0.3	18	Gel strength grade D, dehydration rate 35% after 7 d
	0.6	16	Gel strength grade D, dehydration rate 30% after 7 d
	0.9	13	Gel strength grade D, dehydration rate 30% after 7 d
	1.2	10	Gel strength grade E, dehydration rate 25% after 7 d
	1.5	8	Gel strength grade F, dehydration rate 15% after 7 d

**Table 4:** Plugging strength of gel system.

Sample	Length (cm)	Temp (°C)	Aging (d)	Pressure Gradient (MPa/m)
1 (Water flood)	50	120	1.0	0.24
2 (Water flood)	50	120	3.0	0.22
3 (Water flood)	50	140	1.0	0.21
4 (Water flood)	50	140	3.0	0.19
5 (Gas flood)	50	120	1.0	0.17
6 (Gas flood)	50	120	3.0	0.15
7 (Gas flood)	50	140	1.0	0.14
8 (Gas flood)	50	140	3.0	0.12

## Data Availability

The original contributions presented in this study are included in the article. Further inquiries can be directed to the corresponding author.
